# Race, Ethnicity and Ancestry in Unrelated Transplant Matching for the National Marrow Donor Program: A Comparison of Multiple Forms of Self-Identification with Genetics

**DOI:** 10.1371/journal.pone.0135960

**Published:** 2015-08-19

**Authors:** Jill A. Hollenbach, Aliya Saperstein, Mark Albrecht, Cynthia Vierra-Green, Peter Parham, Paul J. Norman, Martin Maiers

**Affiliations:** 1 Department of Neurology, University of California, San Francisco School of Medicine, San Francisco, California, United States of America; 2 Department of Sociology, Stanford University, Stanford, California, United States of America; 3 Bioinformatics Research, National Marrow Donor Program, Minneapolis, Minnesota, United States of America; 4 Center for International Blood and Marrow Transplant Research, Minneapolis, Minnesota, United States of America; 5 Department of Structural Biology, Stanford University School of Medicine, Stanford, California, United States of America; 6 Department of Microbiology & Immunology, Stanford University School of Medicine, Stanford, California, United States of America; Cincinnati Children's Hospital Medical center, UNITED STATES

## Abstract

We conducted a nationwide study comparing self-identification to genetic ancestry classifications in a large cohort (n = 1752) from the National Marrow Donor Program. We sought to determine how various measures of self-identification intersect with genetic ancestry, with the aim of improving matching algorithms for unrelated bone marrow transplant. Multiple dimensions of self-identification, including race/ethnicity and geographic ancestry were compared to classifications based on ancestry informative markers (AIMs), and the human leukocyte antigen (HLA) genes, which are required for transplant matching. Nearly 20% of responses were inconsistent between reporting race/ethnicity versus geographic ancestry. Despite strong concordance between AIMs and HLA, no measure of self-identification shows complete correspondence with genetic ancestry. In certain cases geographic ancestry reporting matches genetic ancestry not reflected in race/ethnicity identification, but in other cases geographic ancestries show little correspondence to genetic measures, with important differences by gender. However, when respondents assign ancestry to grandparents, we observe sub-groups of individuals with well- defined genetic ancestries, including important differences in HLA frequencies, with implications for transplant matching. While we advocate for tailored questioning to improve accuracy of ancestry ascertainment, collection of donor grandparents’ information will improve the chances of finding matches for many patients, particularly for mixed-ancestry individuals.

## Introduction

The National Marrow Donor Program (NMDP) registry[1] is the repository for data for the highly polymorphic human leukocyte antigen (HLA) genes from over twelve million potential donors for hematopoietic stem cell transplant (HSCT). However, due to changes over time in technology and the costs associated with high resolution HLA genotyping, the registry data for potential donors is often of lower resolution than required for matching with patients. Over ten thousand HLA alleles are currently recognized, with more than 2000 alleles for some genes[2], and worldwide distribution patterns correspond to specific populations with shared demographic history[3]. Accordingly, the NMDP facilitates initial selection of potentially HLA-matched donors by classifying them using their self-identified race/ethnicity from a standardized questionnaire, incorporating this information in a bioinformatics process that projects donors’ most likely high-resolution HLA haplotypes and patient match probabilities[4].

However, racial and ethnic self-identification among Americans is a complex process that draws on information about known ancestry, appearance, how people were raised, where they grew up, whether they experienced discrimination, and by whom; and an individual’s responses can also change over time[5–9]. Some Americans do not report all facets of their ancestry when they are asked to self-identify, even if they know them (e.g., although President Barack Obama often acknowledges his white relatives[10], he identified only as black on the 2010 Census[11]). Others may adjust their responses based on what they assume is the purpose of the data collection[12]. Reporting of geographic origins or ancestry can be equally subjective, as more Americans report common origins (e.g. Irish) than is historically feasible[13]. Thus, the extent to which self-identified race/ethnicity or geographic ancestry will correspond to genetic ancestry is also likely to vary and, *a priori*, it is important to not privilege any one measure of race/ethnicity or ancestry over any other without formal, rigorous testing. Although many studies have examined the relationship between self-identified race/ethnicity and genetic ancestry[14–20], previous work has involved only a single dimension of self-identification without consideration of alternative measures.

Here, we present the first simultaneous assessment of multiple approaches to measuring self-reported ancestry with direct comparison of survey responses with genetic markers, and examine implications for unrelated donor HLA matching in HSCT. We conducted a nationwide study comparing several forms of self-identification to genetic classifications, in a large cohort from the NMDP. We captured multiple facets of self-identification, including race/ethnicity and geographic ancestry. For many Americans these dimensions will yield consistent responses, but for others each measure produces a different response[21]. Our aim was to characterize the extent to which these different self-identification methods correspond not only to each other but also to genetic measures, with the objective of improving current practices for selecting unrelated donors for bone marrow transplants in the United States.

## Materials and Methods

Our cohort includes 1752 individuals who responded positively to a letter of request for participation in the study by mailing back both a cheek swab and a completed paper-and-pencil survey questionnaire. Recipients of the invitation letter were selected randomly with respect to original self-identification from the NMDP donor pool. In order to assess concordance of self-identification with high-resolution HLA genotype, only donors for whom sequence-level data were available for five HLA loci (HLA-A,-B,-C, DRB1, DQB1) were recruited. Because we are interested in improving classification for donors with rare HLA types, we oversampled (2:1) individuals whose HLA genotype is underrepresented in the registry (fewer than 11 copies among 12 million). The National Marrow Donor Program institutional review board approved the study (Study #0292); all subjects gave written informed consent. Response rates by original registry race/ethnicity classification and gender are given in Supplemental materials (S1 Table). The baseline demographics of survey respondents, including the original race/ethnicity classification (based on the existing registry questionnaire; S1 Fig), nativity of respondent, parents and grandparents, age and gender are given in Table 1. Although presented on the original donor form as a separate ethnicity selection, individuals selecting “Hispanic” are treated as equivalent to the major racial categories by the registry, and are considered as such here.

**Table 1:** Survey respondent demographics.

	Total	Female	Male	Mean age
**All respondents**	1752	1324	428	31
**Original registry classification**				
African American	27	22	5	30
White	1414	1090	324	31
Asian or Pacific Islander	65	33	32	30
Hispanic	100	70	30	31
Native American	4	4	0	30
Multi-race	142	105	37	30
**Respondent US born**				
No	100	61	39	33
Yes	1647	1259	388	30
**Parents US Born**				
Neither	159	95	64	31
One	145	114	31	31
Both	1444	1113	331	31
**Grandparents US born**				
None	177	111	66	32
One	28	23	5	29
Two	172	134	38	32
Three	133	102	31	29
Four	1225	944	281	31

In one part of the questionnaire (S2 Fig), we asked respondents how they self-identify with respect to race and ethnicity: *“Mark one or more boxes to show the racial or ethnic group(s) you use to describe yourself*.*”* The list of possible responses included the five major racial categories (American Indian or Alaska Native; Asian; Black or African American; Native Hawaiian or other Pacific Islander; White) recommended by the Office of Management and Budget (OMB) in its 1997 directive[22]. We also included “Hispanic or Latino” and gave the option to mark “other.” A similar instrument is currently being tested for the 2020 Census[12]. In the same section, we asked respondents to tell us their perceived racial classification by others (“*How do other people in this country typically classify you*?*”*). This question was based on an instrument developed by the Center for Disease Control’s Measures of Racism Working Group for the annual Behavioral Risk Factor Surveillance System survey (http://www.cdc.gov/brfss/).

We also asked whether respondents, their parents or their grandparents were born in the United States, followed by a question about their geographic ancestry: “*From what countries or parts of the world did your ancestors come*?*”* These instruments were modeled on items used by the General Social Survey since 1972[23]. Respondents were asked to choose as many categories as necessary to describe their ancestry from a list of countries (e.g., Cuba, Vietnam), regions of the world (e.g., Middle East, Caribbean), and other common ancestries among Americans (“African American” or “North American Indian”). The list of 24 countries combined the most common ancestries reported in the most recent American Community Survey, and the countries from which the most immigrants came to the United States in the last decade. We offered regions of the world to capture countries not specifically included, as well as to provide options for people who might only know general information about family origins. Finally, we asked respondents to provide the specific country, region or ancestry from the same list that applied to each of their four grandparents (“*If possible*, *in the boxes below*, *enter the best origin code for each of your grandparents”)* using numerical codes adjacent to each of the geographic ancestry options. In order to compare this self-identified geographic ancestry to self-identified race/ethnicity and measures of genetic ancestry, we aggregated specific country or region responses into broader categories (S2 Table), in keeping with federal guidelines on racial/ethnic classification in the United States [22] For example, we have combined all Asian geographic ancestries (South, Southeast, East) under one term, “Asian,” as is done by the U.S. Census [24].

A well-characterized panel[25] of 93 ancestry informative markers (AIMs)[26,27] (S3 Table) was genotyped for all respondents using the Sequenom iPLEX assay. Failed single nucleotide polymorphism (SNP) calls underwent a second genotyping using the Taqman assay (ABI). Five percent of the samples served as quality controls and were typed blind in duplicate. All samples and SNPs met standard quality control checks, including fit to expectations under Hardy-Weinberg Equilibrium and less than 10% missingness or failure rates. With the Human Genome Diversity Panel (HGDP) public dataset[28] as a reference, SNP genotypes were used in ancestry estimations with Structure software[29]. Parameters for the Structure run were as follows: k = 4, popflag = 1, burnin = 10,000 and reps = 10,000. The ‘popflag’ option enabled use of the HGDP populations as the training set, while multiple runs indicated that k = 4 clusters allowed the best resolution of broad continental groups (African, European [clustered with Middle Eastern], Asian and Amerindian), facilitated by use of the HGDP populations. A plot of the Structure run showing four distinct clusters is given in S3 Fig We defined subpopulations by race/ethnicity or geographic ancestry responses (e.g., all individuals that selected “Hispanic or Latino” alone or in combination with other race/ethnicity choices; or all individuals reporting any European geographic ancestry). The mean proportion of each genetic ancestry (based on the AIMs) was computed for these subpopulations, and the Wilcoxon Mann-Whitney test used to test differences in distributions of ancestry proportions between groups. All statistical analyses and plotting were performed in the R language and environment for statistical computing[30].

In transplant, the primary genes of interest are HLA. To examine correspondence between self-identification, AIMs and HLA, a Bayesian classifier[31] custom scripted in R was used to assign the most probable continental origin for subjects’ HLA haplotypes. Prior probabilities for marginal distribution of classifications were computed from proportions of the original registry classifications for the cohort. For each subpopulation defined by questionnaire responses, frequencies of African, European. Asian and Amerindian HLA haplotypes were calculated. Correlations between self-identification measures and genetic ancestry proportions (calculated from AIMs) and HLA haplotype origins, as well as correlation between genetic ancestry proportions and HLA origins, were computed using the ‘corr.test’ function in the R package ‘psych.’ For each correlation coefficient, 95% confidence intervals and p-values were computed. All p-values were corrected for multiple tests [32].

Throughout the manuscript, we refer to “race/ethnicity” and “geographic ancestry” to describe measures of self-identification. The term “genetic ancestry” refers to continental ancestry proportions ascertained through AIMs and Structure analysis; and “HLA origin” refers to the continental origin for HLA haplotypes determined via the Bayesian classification. A full description of these terms and their possible values is given in supplemental materials (S4 Table).

## Results

### Self-identification often varies by reporting format

Whereas all subjects self-identified by race/ethnicity, 3% did not provide responses regarding geographic ancestry, and 19% gave what could be considered inconsistent responses for these two questions (S5 Table). Approximately half of the inconsistent responses reported American Indian ancestry without identifying their race/ethnicity as American Indian. Most often, lack of concordance between geographic ancestry and race/ethnicity reporting occurs when individuals acknowledge particular geographic ancestries but do not explicitly identify with a corresponding racial/ethnic group. Less common is race/ethnicity self-identification without reporting a corresponding geographic ancestry. Consequently, many more individuals reported multiple geographic ancestries (19%) than multiple race/ethnicities (7%). The vast majority of individuals who reported a single race/ethnicity also reported that other people typically perceive them the same way. However, consistent with evidence from other nationally representative surveys [9], the majority of individuals who reported multiple race/ethnicities reported that other people typically perceive them as White.

### Correspondence of self-identification measures with genetic ancestry and HLA origin

No measure of self-identification shows complete correspondence with any specific pattern of genetic ancestry, underscoring the unclear boundaries and imprecise nature of these categorizations, which are often presumed to represent separate and identifiable groups. We find evidence of European genetic ancestry among nearly all sub-populations, as expected in a U.S. cohort (Table 2). However, the proportion of European genetic ancestry ranges broadly, with lowest average values observed among individuals who identify their race/ethnicity Asian. African genetic ancestry proportions range from 20–95% for individuals who identify their race/ethnicity as “Black or African American,” with the remaining component largely European. For individuals who self-identify their race/ethnicity as “Hispanic or Latino” we observe mean European and Amerindian genetic ancestries of 71% and 21%, respectively, but the defining feature of these classifications is that the proportion of each genetic ancestry varies broadly between individuals even when they self-identify the same way.

**Table 2:** Mean genetic ancestry proportions determined via ancestry informative markers and HLA haplotype origin frequencies for subpopulations defined by reported race/ethnicity or geographic ancestry.

	N	Mean genetic ancestry proportion					HLA haplotype origin				
		African	European	Asian		Amerindian	African American	European	Asian	Amerindian	Multi-origin*
**Race/Ethnicity Self-ID: Single choice respondents**	1624										
**Black or African American**	27	0.70	0.27		0.03	0.01	63%	11%	4%	-	22%
**White**	1443	0.00	0.99		0.00	0.00	-	82%	-	-	17%
**Asian**	64	0.01	0.22		0.76	0.01	-	-	97%	-	3%
**Hispanic or Latino**	71	0.05	0.63		0.05	0.28	-	13%	1%	52%	34%
**American Indian or Alaska Native**	2	0.00	0.99		0.01	0.00	-	50%	-	-	50%
**Native Hawaiian or other Pacific Islander**	2	0.01	0.00		0.97	0.02	-	-	100%	-	-
**Geographic ancestry: Single origin respondents**	1373										
**Africa or African American**	15	0.71	0.25		0.03	0.02	67%	13%	7%	-	13%
**Europe**	1236	0.00	0.99		0.00	0.00	-	82%	-	-	17%
**Asia/Pacific**	63	0.01	0.21		0.77	0.01	-	-	97%	-	3%
**North American Indian**	6	0.00	0.91		0.01	0.08	-	20%	-	-	80%
**Latin America**	36	0.04	0.51		0.07	0.38	-	-	-	72%	28%
**Caribbean**	17	0.19	0.71		0.03	0.07	6%	-	6%	12%	77%
**Race/Ethnicity Self-ID: Multiple choice respondents**	124	0.04	0.84		0.06	0.05	5%	48%	6%	6%	35%
**Black or African American**	13	0.36	0.60		0.03	0.02	54%	8%	-	-	39%
**White**	120	0.03	0.85		0.06	0.05	3%	48%	7%	6%	36%
**Asian**	18	0.00	0.66		0.31	0.02	-	22%	33%	6%	39%
**Hispanic or Latino**	46	0.03	0.84		0.03	0.11	4%	37%	-	13%	46%
**American Indian or Alaska Native**	53	0.04	0.91		0.02	0.03	6%	66%	2%	4%	23%
**Native Hawaiian or other Pacific Islander**	5	0.01	0.71		0.23	0.05	-	20%	60%	-	20%
**Geographic ancestry: Multiple origin respondents**	379	0.04	0.89		0.03	0.04	4%	63%	3%	6%	25%
**Africa or African American**	27	0.40	0.57		0.02	0.02	41%	22%	-	-	37%
**Europe**	318	0.02	0.91		0.03	0.04	2%	64%	4%	5%	25%
**Asia/Pacific**	25	0.01	0.70		0.28	0.02	-	28%	36%	4%	32%
**North American Indian**	49	0.04	0.91		0.02	0.03	2%	72%	1%	3%	23%
**Latin America**	68	0.05	0.78		0.02	0.15	6%	40%	-	22%	32%
**Caribbean**	21	0.19	0.75		0.04	0.02	14%	48%	5%	-	33%
**Classification by others**											
**Black or African American**	31	0.71	0.25		0.03	0.01	68%	10%	-	-	23%
**White**	1545	0.00	0.98		0.01	0.01	-	80%	-	-	18%
**Asian**	70	0.01	0.24		0.74	0.01	-	7%	90%	1%	1%
**Hispanic or Latino**	73	0.05	0.63		0.05	0.28	1%	10%	1%	52%	36%
**American Indian or Alaska Native**	6	0.01	0.74		0.11	0.14	-	67%	-	-	33%
**Native Hawaiian or other Pacific Islander**	3	0.01	0.67		0.31	0.01	-	33%	33%	-	33%
**Other**	23	0.10	0.71		0.13	0.05	9%	26%	26%	4%	35%

* All individuals have two HLA haplotypes. Multi-origin haplotype classification indicates that one of the individual’s haplotypes is closely associated with one continental origin while the other haplotype is associated with a different continental origin.

Across all classifications and question formats, we find very high correlation between the mean proportion of African, European, Asian and Amerindian genetic ancestry in each subpopulation, and the frequency of the respective HLA haplotypes origins in those individuals (r = 0.97, 0.79, 0.97, 0.95, respectively; Table 2). This demonstrates concordance of population level genetic ancestry assessed via AIMs and HLA origin. The strongest correlation between any two measures is between self-reported Asian race/ethnicity and self-reported Asian geographic ancestry (r = 0.94; CI = 0.93–0.94; p<10^−15^), and each of these measures is similarly correlated with Asian HLA origin and genetic ancestry (Table 3). Sub-Saharan African geographic ancestry and racial/ethnic self-identification as “Black or African American” are also highly correlated (r = 0.83; CI = 0.81–0.84; p<10^−15^), but the latter tracks somewhat more closely with African HLA origin and genetic ancestry (Table 3). We find lower, yet significant correspondence between European geographic ancestry and self-identification as White (r = 0.68; CI = 0.66–0.71; p<10^−15^), with race/ethnicity being slightly more predictive of European HLA origin and genetic ancestry (Table 3). Individuals reporting Latin American geographic ancestry (e.g., Mexico, Guatemala, Central or South America) tend to self-identify their race/ethnicity as “Hispanic or Latino” (r = 0.76; CI = 0.73–0.77; p<10^−15^) and have Amerindian HLA origin and genetic ancestry (Table 3), while those reporting Caribbean geographic ancestry (e.g., Puerto Rico) do not identify their race/ethnicity as Hispanic as frequently (r = 0.37; CI = 0.33.0.41; p<10^−15^), have much lower proportions of Amerindian genetic ancestry, and are less likely to have Amerindian HLA origin (Table 3). North American Indian geographic ancestry is correlated with reporting multiple race/ethnicities or ancestries (r = 0.80; CI = 0.78–0.82; p<10^−15^), but not any specific genetic ancestry.

**Table 3:** Correlation between self-identification measures and HLA origins and genetic ancestry.

Self-identification measure	HLA origins				Genetic ancestry (AIMs)			
	African	European	Asian	Amerindian	African	European	Asian	Amerindian
*Race/ethnicity*								
Black or African American	0.71 (0.68–0.74)				0.90 (0.89–0.91)			
White		0.74 (0.72–0.77)				0.77 (0.74–0.79)		
Asian			0.84 (0.82–0.85)				0.84 (0.82–0.86)	
Hispanic or Latino				0.58 (0.54–0.62)				0.66 (0.63–0.69)
Native Hawaiian or other Pacific Islander			0.14 (0.09–0.19)				0.15 (0.10–0.21)	
*Geographic ancestry*								
African or African American	0.69 (0.66–0.72)				0.82 (0.80–0.83)			
European		0.72 (0.69–0.75)				0.75 (0.73–0.77)		
Asian			0.83 (0.81–0.85)				0.83 (0.81–0.85)	
Latin American				0.54 (0.50–0.58)				0.69 (0.66–0.72)
Caribbean				0.13 (0.07–0.18)	0.20 (0.14–0.25)			
*Grandparents ancestry*								
African or African American	0.72 (0.69–0.74)				0.86 (0.85–0.88)			
European		0.77 (0.75–0.79)				0.81 (0.79–0.83)		
Asian			0.88 (0.87–0.89)				0.89 (0.88–0.90)	
Latin American				0.62 (0.59–0.66)				0.80 (0.78–0.82)
Caribbean	0.10 (0.05–0.16)			0.17 (0.120.23)	0.18 (0.12–0.23)			

The correlation coefficient (r) is given with 95% confidence intervals (parentheses). Only statistically significant (p<0.05 after correction) values are shown.

While distinguishing between reported Latin American and Caribbean geographic ancestry affords advantage over Hispanic racial/ethnic identification in representing genetic ancestry, other geographic ancestries are more weakly related to comparable genetic classifications. For example, 3% of respondents reported European geographic ancestry in addition to other origins, but did not identify as White with respect to race/ethnicity (n = 52). Of those, nearly two-thirds self-identified their race/ethnicity as Hispanic (n = 28) or African American (n = 4); however, mean European genetic ancestry for self-identified Hispanics and African Americans who reported European geographic ancestry (0.73 and 0.34, respectively) is not statistically significantly higher than that for individuals with the same self-identified race/ethnicity who do not report European geographic ancestry (0.63 and 0.27).

### Gender differences in reporting consistency have implications for HLA matching

In some cases when geographic ancestry yields less consistent classifications relative to genetic ancestry than those based on race/ethnicity, we find the differences also vary by gender. More than 10 percent of our cohort reported North American Indian geographic ancestry but did not self-identify as American Indian with respect to race/ethnicity (n = 189). The mean Amerindian genetic ancestry proportion (0.018) for these individuals is similar to those not reporting North American Indian ancestry (0.019). Strikingly, significantly more women (n = 164; 12%) than men (n = 25; 6%) report North American Indian ancestry in addition to another geographic ancestry without also identifying their race/ethnicity as American Indian (p< 0.001). Further, and of most importance in transplant matching, selection of more than one race/ethnicity or geographic ancestry by men is much more predictive of multi-origin HLA than multi-origin reporting among women, even though men are less likely to identify with multiple race/ethnicities or geographic ancestries. For example, 28% of men who self-identify with two or more race/ethnicity groups have multi-origin HLA haplotypes; in contrast, just 9% of women selecting more than one race/ethnicity group have multi-origin HLA haplotypes (p<0.05).

### Grandparents’ geographic ancestry most closely correlated with HLA haplotype origins

**Fig 1:**
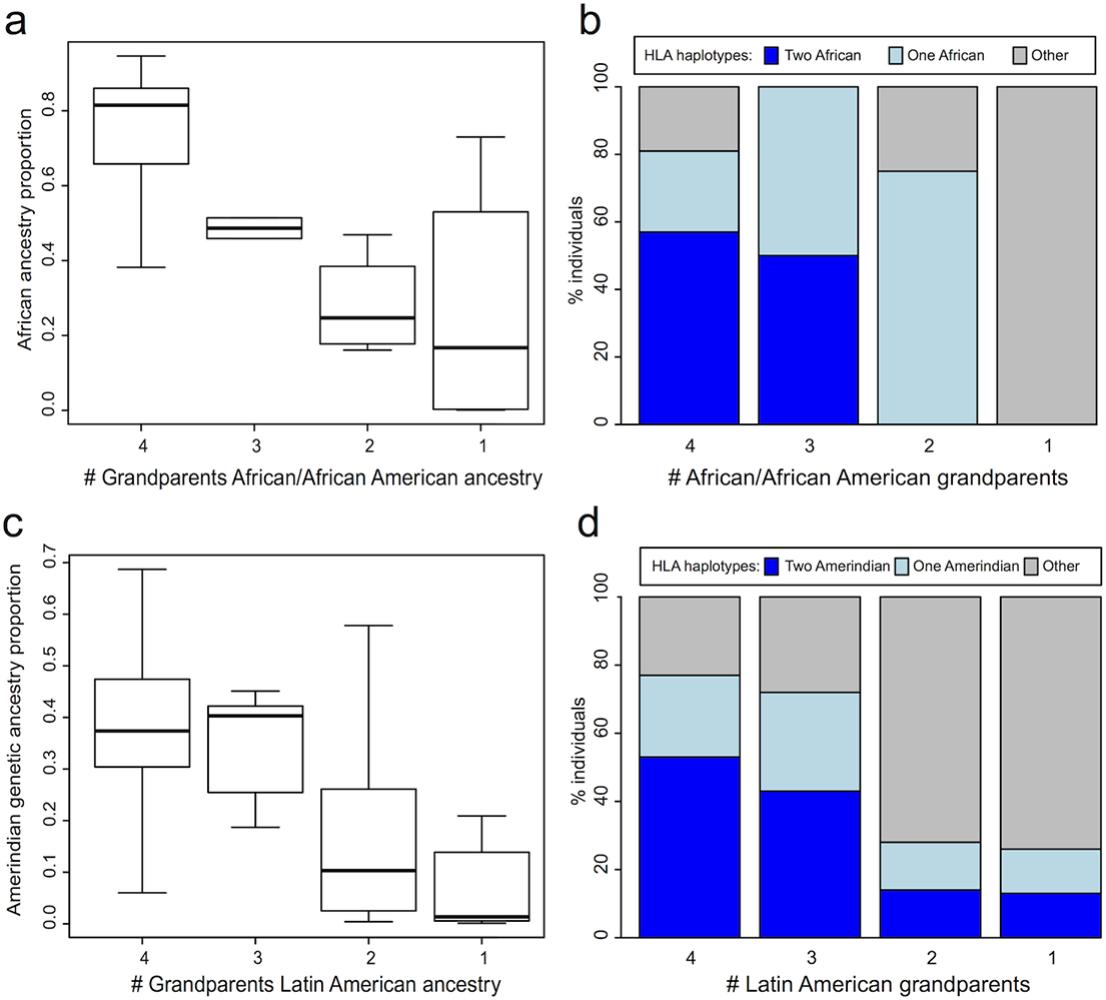
African and Amerindian genetic ancestry proportions (a,c) and HLA origin frequencies (b,d) are correlated with the number of grandparents with reported African and Latin American ancestry, respectively. Genetic ancestry proportions were estimated from AIMs data using Structure and the HGDP reference set. For this analysis, k = 4, reflecting broad continental ancestry. For each subpopulation defined by the number of respondents’ grandparents with Sub-Saharan African/African American or Latin American ancestry, the percentage of individuals with zero, one or two African or Amerindian HLA haplotypes, respectively, was calculated. Individuals reporting one, two, three or four African-ancestry grandparents: n = 4; 4; 2; 21, respectively. Individuals reporting one, two, three or four Latin American-ancestry grandparents: n = 16; 21; 7; 38, respectively.

In contrast to reporting generic family origins, when respondents select origins for specific grandparents the resulting classification using the number of grandparents with a given geographic ancestry identifies sub-groups of respondents with more uniform genetic backgrounds. Sub-classification by the number of grandparents with Sub-Saharan African or African American ancestry, for example, shows a clear pattern of increasing African genetic ancestry proportions with increasing number of reported African-ancestry grandparents (Fig 1A). The substructure is mirrored in HLA origin frequencies, identifying groups of individuals with different combinations of HLA haplotypes. This result has important implications for the transplant match algorithm’s assignment of likely high resolution HLA haplotypes. For example, well over half of the individuals who reported four African-ancestry grandparents have two African HLA haplotypes; in contrast, three of the four individuals who reported two African-ancestry grandparents have only one African HLA haplotype (Fig 1B). The results are similar for individuals who reported grandparents from Latin American origins, with respect to Amerindian genetic ancestry proportions and HLA (Fig 1C and 1D). These findings hold for European and Asian genetic ancestry proportions and HLA origin frequencies as well (not shown), demonstrating the value in collecting grandparents’ ancestry for all individuals as a substantial improvement to current racial/ethnic self-identification practices for prospective donors.

## Discussion

In medical practice and biomedical research, subjects’ self-identified race/ethnicity is frequently collected[33–35], often serving as a proxy for genetic ancestry. At the same time, there is ongoing discourse among physicians, researchers and the public about how best to collect this critical information[36,37], the relationship between racial classification and genetic ancestry [38–46], its possible confounding role in biomedical research[16,47] and its application in health care settings. Using AIMs, many studies have shown good correlation between genetics and self-identification for some racial and ethnic categories in the United States[16,18], but a limited correspondence for individuals who identify as Hispanic or multi-racial[14,19,48]. Genetic clustering of worldwide human populations[27] suggests that identification according to geographic ancestry may be more biologically relevant than the standard race/ethnicity categories used in federal data collection and reporting. Yet, many remain wary of assumptions about inherent difference and internal homogeneity that have often characterized the use of race and ancestry identification in biomedical fields[49,50]. Too often, however, these discussions take place at an abstract level–about what should and should not be done in general, or under ideal circumstances. Equally important is careful examination of the practicality and relevance of different measures of ancestry identification for specific medical applications where these data are currently relied upon for patient care: such as matching donors and recipients for transplant.

With the aim of improving matching algorithms for bone marrow transplant, this study sought to determine how various measures of self-identification intersect with genetic ancestry. Our finding that nearly one in five respondents provided answers inconsistent between reported race/ethnicity and geographic ancestry highlights the subjective nature of self-identification. In comparing measures of self-identification, we do not mean to imply that we think responses should always be consistent across measures or that one type of response is more “correct” than another. Rather, we want to underscore how different measures of self-identification reveal different information. Moreover, it is apparent that there is no clear ‘winner’ in the complex process of self-identification in the U.S. with respect to genetic ancestry; best practices for collecting this information for some populations may be ineffective for others. Unraveling the myriad historical, political and social factors contributing to these differences among measures of self-identification, and between self-reported and genetic ancestry measures, is beyond the scope of this work. Instead, our results point to the need for more research to identify the specific nature of this variation and refine how we collect and interpret measures of race/ethnicity and geographic ancestry in medical contexts.

As expected, in certain cases, geographic ancestry reporting more closely corresponds to genetic ancestry compared to self-identification using standard race/ethnicity categories. While often classed together as “Hispanic” by registries and in much biomedical research, individuals reporting Latin American origins display markedly different proportions of African and Amerindian genetic ancestry than those who report Caribbean origins[51,52]; our results are concordant with these findings and, most important for the registry, mirrored in the frequency of HLA subclasses. In contrast, reported European geographic ancestry provides limited additional insight into genetic ancestry proportions for most. The reporting of North American Indian geographic ancestry is also generally not consistent with genetic classifications. These individuals may be reporting very distant ancestry that has survived in family lore, or this may reflect people selecting symbolic responses not based on specific knowledge of family origins[6,13]. That most respondents reporting African American racial identification did not report sub-Saharan African geographic ancestry also suggests that geographic ancestry is not a form of self-identification that resonates with everyone.

Starting in 2000, the U.S. Census questionnaire allowed respondents to select more than one self-identified race. In 2010, over 9 million Americans identified with two or more races, a 32% increase from the previous decade, and a substantial fraction of our cohort reports multiple race/ethnicities or geographic ancestries. Likewise, individuals who self-identify as Hispanic now comprise the largest racial/ethnic group within the United States after non-Hispanic Whites[53,54]. In our study, grandparents’ ancestry is the most informative piece of data with respect to more precisely characterizing genetic ancestry for individuals from populations long recognized as highly heterogeneous[48,52], and collection of this information should improve the efficiency and reliability of finding HLA-identical donors for many patients. Although not currently collected by the registry, in the health care setting, or for most biomedical research, in our sample >70% respondents provided a geographic ancestry for all four grandparents. Because reporting for each grandparent was limited to only one origin or ancestry, these responses may be particularly salient, deriving from direct knowledge of a close relative. We suggest that registries consider collecting these valuable data routinely. In contrast, when asked to “check all that apply” to describe one’s family origins in general, the threshold for claiming particular geographic ancestries may be much lower, and the responses are not constrained to four with equal weight. However, given that not all respondents are able to provide data for their grandparents, this measure should be used in combination with others. Providing the means for individuals to quantify or otherwise describe the relative salience of their ancestries may also be of value in future studies.

A major limitation here is over-representation of individuals who self-identify as White compared to the current U.S. population and an over-representation of individuals with European-origin HLA types compared to the overall makeup of the registry; additional work in this area will profit from study of larger samples of subpopulations with non-European origins and ancestries. We focused on low-frequency HLA haplotypes in this study under a working premise that individuals with complex ancestries, which might not be fully captured by the current registry questionnaire, were more likely to have rare HLA types. We hypothesized that alternate forms of self-identification might provide improved matching for these individuals. Further, we sought a cohort with diverse ancestry, but did not wish to base our sampling strategy on current registry race/ethnicity classifications. However, the survey response rates varied by gender and original registry race/ethnicity classifications yielding a less diverse sample, and results must be interpreted within this context. Importantly, 62% of respondents were women whose original registry classification was White, compared to only 35% of those individuals who received the initial letter inviting participation in the study. This group had the highest participation rate overall (10%), while the lowest participation rates were among African American men (<1%). Additionally, genetic ancestry proportions estimated in this study are sensitive to the markers examined and population samples used as background, and thus resolution of these proportions will be somewhat limited for some populations. Nevertheless, the strong correlation between AIMs and HLA in our study supports the notion that AIMs can track with medically important variation. Finally, our analysis did not find an association between the frequency of individuals’ HLA types in the registry with any specific self-identification measure or inconsistency in the survey responses. However, given our finding that classification is variable depending on the format for self-identification, this pilot study benefited by not sampling according to any *a priori* racial or ethnic classification scheme.

Despite cultural and scientific controversy, collection of data regarding race, ethnicity and ancestry will likely continue to play a role in medicine and biomedical research, compelling the need to understand better how patterns of self-identification interact with genetic classifications and health outcomes for all Americans. Although personalized medicine and whole-genome sequencing may eventually circumvent the need for ancestry self-identification in some settings, at present this approach is cost-prohibitive for donor registries, and impossible in other cases where biological samples are not collected. When relying on self-reporting, it is important to consider both the political origins of our now-standard classification schemes [55–57] and that social factors can make it difficult to claim some racial/ethnic identities or geographic ancestries and perhaps too easy to claim others. The challenge for medicine is to understand when self-identification facilitates diagnosis and treatment and when certain types of self-reporting might be confounding. Our results will form the basis for further inquiries into self-identification measures relevant to the donor registry that best reflect variation in HLA in order to improve transplant matching, with broader implications for medical practice and biomedical research.

## Supporting Information

S1 Fig The original race/ethnicity questions completed by respondents at the time of donor registration. The registry collapsed all responses into the broad categories listed at the top of each answer section. Individuals selecting “Hispanic” were assigned to a separate category.
(TIFF)

S2 Fig The ancestry survey questionnaire completed by all respondents. (TIFF)

S3 Fig Plot of the Structure run used to ascertain genetic ancestry proportions from AIMs. For this run, k = 4, popflag = 1, burnin = 10,000 and reps = 10,000. Individuals are represented in the following order from left to right: HGDP African populations, HGDP Middle Eastern populations, HGDP European populations, HGDP Central/South Asian populations, HGDP East Asian populations, HGDP Amerindian populations, HGDP Oceanic populations, study respondents. Broad continental ancestries are color-coded as follows: African = yellow; European = green; Asian = red; Amerindian = blue.
(ZIP)

S1 Table Survey response rates by original registry race classification and gender. The number of individuals in each category (recipients of initial contact letter requesting participation; respondents in the study) and the percentage of the total in that category is given in parentheses. Response rates are calculated as Respondents/Initial contact in each category, as well as combined (“All”) for both genders. The ratio of female to male respondents in each registry classification group is given to the right.
(DOCX)

S2 Table Mapping of responses for geographic ancestries to broad ancestries that are considered the basis for major racial and ethnic classifications in the United States. (DOCX)

S3 Table The panel of ancestry informative markers examined in the study. (DOCX)

S4 Table Description of ancestry terminology used in the study. (DOCX)

S5 Table Consistency between self-identified race/ethnicity and geographic ancestry. (DOCX)
