# Supplementary figures and images for: Race, Ethnicity and Ancestry in Unrelated Transplant Matching for the National Marrow Donor Program: A Comparison of Multiple Forms of Self-Identification with Genetics
Source: PLoS One. 2015 Aug 19;10(8):e0135960. doi: 10.1371/journal.pone.0135960 (PMC4545604; doi:10.1371/journal.pone.0135960)

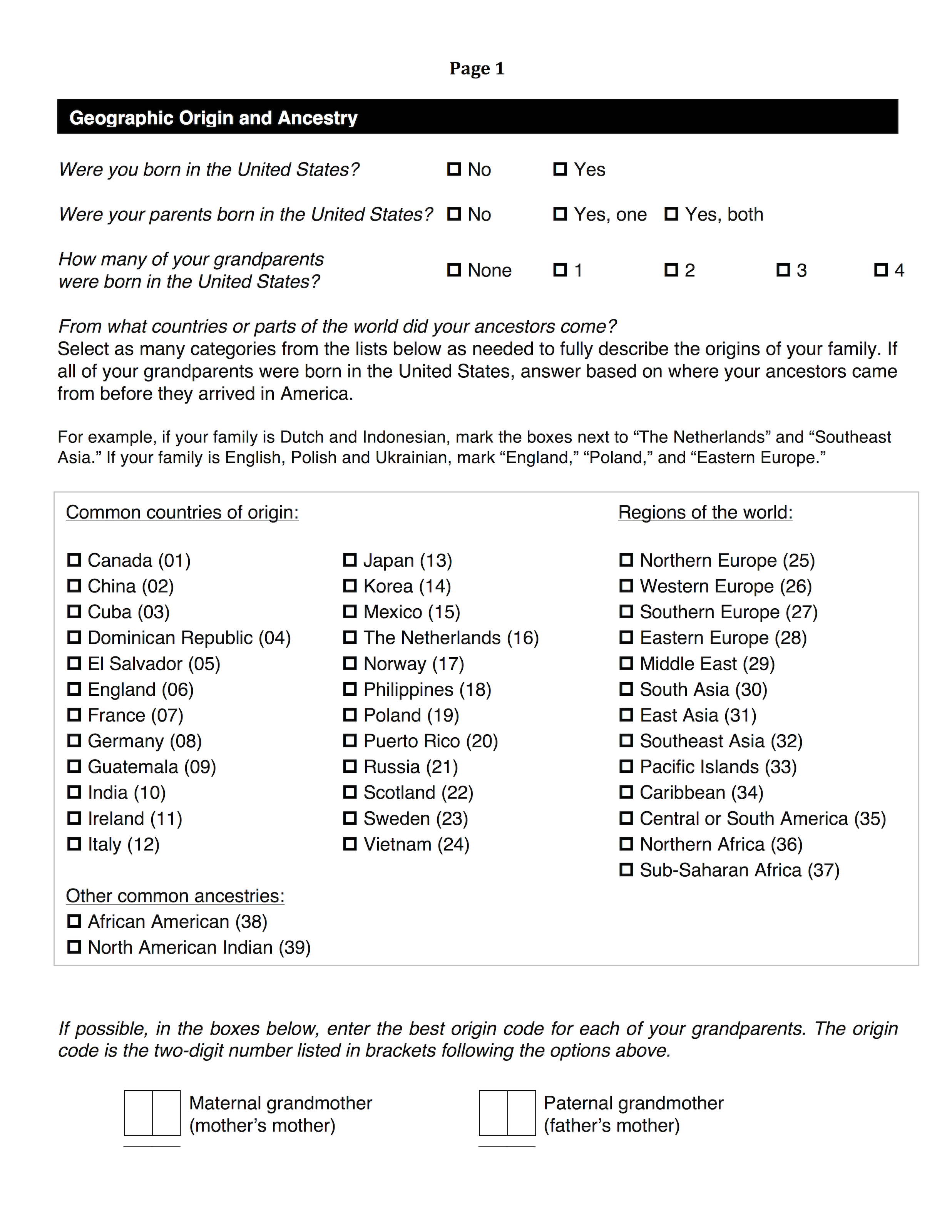

Supplement: S2 Fig — (TIFF) [file pone.0135960.s002.tiff]
