# Supplementary material for: Race, Ethnicity and Ancestry in Unrelated Transplant Matching for the National Marrow Donor Program: A Comparison of Multiple Forms of Self-Identification with Genetics
Source: PLoS One. 2015 Aug 19;10(8):e0135960. doi: 10.1371/journal.pone.0135960 (PMC4545604; doi:10.1371/journal.pone.0135960)
Supplement: S1 Table — The number of individuals in each category (recipients of initial contact letter requesting participation; respondents in the study) and the percentage of the total in that category is given in parentheses. Response rates are calculated as Respondents/Initial contact in each category, as well as combined (“All”) for both genders. The ratio of female to male respondents in each registry classification group is given to the right. (DOCX) [file pone.0135960.s004.docx]

**S1 Table. Survey response rates by original registry race/ethnicity classification and gender.** The number of individuals in each category (recipients of initial contact letter requesting participation; respondents in the study) and the percentage of the total in that category is given in parentheses. Response rates are calculated as Respondents/Initial contact in each category, as well as combined (“All”) for both genders. The ratio of female to male respondents for each race/ethnicity is given to the right.

|  | **Initial contact** | | **Respondents** | | **Response rates** | | |  |
| --- | --- | --- | --- | --- | --- | --- | --- | --- |
| **Original classification** | Female | Male | Female | Male | Female | Male | All | Female:Male |
| African American or Black | 1093 (0.036) | 804 (0.027) | 22 (0.013) | 5 (0.003) | 0.020 | 0.006 | 0.014 | 3.24 |
| White | 10634 (0.354) | 6052 (0.202) | 1090 (0.622) | 324 (0.185) | 0.103 | 0.054 | 0.085 | 1.91 |
| Asian or Pacific Islander | 1549 (0.052) | 1937 (0.065) | 33 (0.019) | 32 (0.018) | 0.021 | 0.017 | 0.019 | 1.29 |
| Hispanic | 2821 (0.094) | 1980 (0.066) | 70 (0.040) | 30 (0.017) | 0.025 | 0.015 | 0.021 | 1.64 |
| North American Indian | 67 (0.002) | 45 (0.002) | 4 (0.002) | 0 | 0.060 | 0.000 | 0.036 | NA |
| Multi-race | 1791 (0.060) | 1212 (0.040) | 105 (0.060) | 37 (0.021) | 0.059 | 0.031 | 0.047 | 1.92 |
