# Supplementary material for: Race, Ethnicity and Ancestry in Unrelated Transplant Matching for the National Marrow Donor Program: A Comparison of Multiple Forms of Self-Identification with Genetics
Source: PLoS One. 2015 Aug 19;10(8):e0135960. doi: 10.1371/journal.pone.0135960 (PMC4545604; doi:10.1371/journal.pone.0135960)
Supplement: S2 Table — (DOCX) [file pone.0135960.s005.docx]

Table S2. Mapping of detailed family origin responses to broader ancestry categories for comparison with major racial and ethnic classifications in the United States.

| Reported family origin | Geographic ancestry |
| --- | --- |
| Canada | European |
| China | Asian |
| Cuba | Caribbean |
| Dominican Republic | Caribbean |
| El Salvador | Latin America |
| England | European |
| France | European |
| Germany | European |
| Guatemala | Latin America |
| India | Asian |
| Ireland | European |
| Italy | European |
| Japan | Asian |
| Korea | Asian |
| Mexico | Latin America |
| The Netherlands | European |
| Norway | European |
| Philippines | Asian |
| Poland | European |
| Puerto Rico | Caribbean |
| Russia | European |
| Scotland | European |
| Sweden | European |
| Vietnam | Asian |
| Northern Europe | European |
| Western Europe | European |
| Southern Europe | European |
| Eastern Europe | European |
| Middle East | European |
| South Asia | Asian |
| East Asia | Asian |
| Southeast Asia | Asian |
| Pacific Islands | Pacific |
| Caribbean | Caribbean |
| Central or South America | Latin America |
| Northern Africa | European |
| Sub-Saharan Africa | African |
| African American | African |
