# Supplementary material for: Race, Ethnicity and Ancestry in Unrelated Transplant Matching for the National Marrow Donor Program: A Comparison of Multiple Forms of Self-Identification with Genetics
Source: PLoS One. 2015 Aug 19;10(8):e0135960. doi: 10.1371/journal.pone.0135960 (PMC4545604; doi:10.1371/journal.pone.0135960)
Supplement: S3 Table — (DOCX) [file pone.0135960.s006.docx]

Table S3. The panel of ancestry informative markers examined in the study.

| Marker* | Chromosome |
| --- | --- |
| rs1040404 | 1 |
| rs12130799 | 1 |
| rs1325502 | 1 |
| rs1407434 | 1 |
| rs2986742 | 1 |
| rs3118378 | 1 |
| rs316873 | 1 |
| rs3737576 | 1 |
| rs4908343 | 1 |
| rs647325 | 1 |
| rs7554936 | 1 |
| rs10496971 | 2 |
| rs13400937 | 2 |
| rs260690 | 2 |
| rs2627037 | 2 |
| rs4666200 | 2 |
| rs4670767 | 2 |
| rs7421394 | 2 |
| rs798443 | 2 |
| rs10510228 | 3 |
| rs12629908 | 3 |
| rs1513181 | 3 |
| rs2030763 | 3 |
| rs6548616 | 3 |
| rs734873 | 3 |
| rs9809104 | 3 |
| rs9845457 | 3 |
| rs1369093 | 4 |
| rs2702414 | 4 |
| rs385194 | 4 |
| rs7657799 | 4 |
| rs12657828 | 5 |
| rs316598 | 5 |
| rs6422347 | 5 |
| rs6451722 | 5 |
| rs870347 | 5 |
| rs1040045 | 6 |
| rs1871428 | 6 |
| rs2397060 | 6 |
| rs2504853 | 6 |
| rs4463276 | 6 |
| rs7745461 | 6 |
| rs10236187 | 7 |
| rs2330442 | 7 |
| rs32314 | 7 |
| rs4717865 | 7 |
| rs6464211 | 7 |
| rs705308 | 7 |
| rs731257 | 7 |
| rs7803075 | 7 |
| rs10108270 | 8 |
| rs12544346 | 8 |
| rs1471939 | 8 |
| rs3943253 | 8 |
| rs7844723 | 8 |
| rs10513300 | 9 |
| rs2073821 | 9 |
| rs2306040 | 9 |
| rs4746136 | 10 |
| rs4918842 | 10 |
| rs10839880 | 11 |
| rs11227699 | 11 |
| rs1837606 | 11 |
| rs2946788 | 11 |
| rs948028 | 11 |
| rs214678 | 12 |
| rs2416791 | 12 |
| rs772262 | 12 |
| rs7997709 | 13 |
| rs9319336 | 13 |
| rs9522149 | 13 |
| rs9530435 | 13 |
| rs1760921 | 14 |
| rs1950993 | 14 |
| rs200354 | 14 |
| rs2357442 | 14 |
| rs3784230 | 14 |
| rs12439433 | 15 |
| rs8035124 | 15 |
| rs4781011 | 16 |
| rs4984913 | 16 |
| rs818386 | 16 |
| rs10512572 | 17 |
| rs11652805 | 17 |
| rs2125345 | 17 |
| rs7238445 | 18 |
| rs874299 | 18 |
| rs3745099 | 19 |
| rs8113143 | 19 |
| rs3907047 | 20 |
| rs1296819 | 22 |
| rs4821004 | 22 |
| rs5768007 | 22 |

*All ancestry informative markers are single nucleotide polymorphisms, numbered in accordance with the National Center for Biotechnology Information.
