# Supplementary material for: Race, Ethnicity and Ancestry in Unrelated Transplant Matching for the National Marrow Donor Program: A Comparison of Multiple Forms of Self-Identification with Genetics
Source: PLoS One. 2015 Aug 19;10(8):e0135960. doi: 10.1371/journal.pone.0135960 (PMC4545604; doi:10.1371/journal.pone.0135960)
Supplement: S4 Table — (DOCX) [file pone.0135960.s007.docx]

**S4 Table. Description of ancestry terminology used in the study.**

| **Term** | **Description** | **Values** |
| --- | --- | --- |
| Race/ethnicity | Categories that individual uses to describe themselves based on federal classification guidelines | American Indian or Alaska Native; Asian; Black or African American; Hispanic or Latino; Native Hawaiian or other Pacific Islander; White; Other |
| Geographic ancestry | Countries or regions of origin assigned by individual to describe their family origins (non-specific) | Canada; China; Cuba; Dominican Republic; El Salvador; England; France; Germany; Guatemala; India; Ireland; Italy; Japan; Korea; Mexico; The Netherlands; Norway ; Philippines; Poland; Puerto Rico; Russia; Scotland; Sweden; Vietnam; Northern Europe; Western Europe; Southern Europe; Eastern Europe; Middle East; South Asia; East Asia; Southeast Asia; Pacific Islands; Caribbean; Central or South America; Northern Africa; Sub-Saharan Africa |
| Grandparents ancestry | Country or region of origin assigned by individual to specific grandparents | Canada; China; Cuba; Dominican Republic; El Salvador; England; France; Germany; Guatemala; India; Ireland; Italy; Japan; Korea; Mexico; The Netherlands; Norway ; Philippines; Poland; Puerto Rico; Russia; Scotland; Sweden; Vietnam; Northern Europe; Western Europe; Southern Europe; Eastern Europe; Middle East; South Asia; East Asia; Southeast Asia; Pacific Islands; Caribbean; Central or South America; Northern Africa; Sub-Saharan Africa |
| Genetic ancestry | Ancestry proportion derived from AIMs | African; European; Asian; Amerindian |
| HLA origin | Likely continental origin for an individual's HLA haplotypes | African; European; Asian; Amerindian |
