# Supplementary material for: Race, Ethnicity and Ancestry in Unrelated Transplant Matching for the National Marrow Donor Program: A Comparison of Multiple Forms of Self-Identification with Genetics
Source: PLoS One. 2015 Aug 19;10(8):e0135960. doi: 10.1371/journal.pone.0135960 (PMC4545604; doi:10.1371/journal.pone.0135960)
Supplement: S5 Table — (DOCX) [file pone.0135960.s008.docx]

**S5 Table. Consistency between self-identified race/ethnicity and geographic ancestry.**

|  | Total reported race/ethnicity | % inconsistent with geographic ancestry reporting* |
| --- | --- | --- |
| Black or African American | 40 | 14% |
| White | 1563 | 4% |
| Asian | 82 | 0% |
| Hispanic or Latino | 117 | 7% |
| North American Indian | 55 | 15% |
| Native Hawaiian/Pacific Islander | 7 | 71% |
| All race/ethnicities | 1864 | 5% |
|  |  |  |
|  |  |  |
|  | Total reported geographic ancestry | % inconsistent with race/ethnicity reporting* |
| Africa or African American | 42 | 19% |
| Europe | 1554 | 3% |
| Asia | 85 | 6% |
| Latin America or Caribbean | 138 | 21% |
| North American Indian | 236 | 80% |
| Pacific Islands | 5 | 60% |
| All ancestries | 2060 | 19% |

___________________________________________________________________________________________

*For each race/ethnicity reported: Black or African American, White, Asian, Hispanic or Latino, North American Indian or Native Hawaiian/Pacific Islander, individuals’ responses were compared to those for the corresponding geographic ancestry: Africa or African American, Europe, Asia, Latin America or Caribbean, North American Indian or Pacific Islands, respectively.
